# Characterising seizures in anti-NMDA-receptor encephalitis with dynamic causal modelling

**DOI:** 10.1016/j.neuroimage.2015.05.064

**Published:** 2015-09

**Authors:** Gerald K. Cooray, Biswa Sengupta, Pamela Douglas, Marita Englund, Ronny Wickstrom, Karl Friston

**Affiliations:** a Wellcome Trust Centre for Neuroimaging, Institute of Neurology, University College London, UK; b Clinical Neurophysiology, Karolinska University Hospital, Stockholm, Sweden; c Neuropediatric Unit, Department of Women's and Children's Health, Karolinska Institutet, Sweden

**Keywords:** Anti-NMDA-R encephalitis, EEG, Dynamical causal modelling (DCM), Seizures

## Abstract

We characterised the pathophysiology of seizure onset in terms of slow fluctuations in synaptic efficacy using EEG in patients with anti-N-methyl-d-aspartate receptor (NMDA-R) encephalitis. EEG recordings were obtained from two female patients with anti-NMDA-R encephalitis with recurrent partial seizures (ages 19 and 31). Focal electrographic seizure activity was localised using an empirical Bayes beamformer. The spectral density of reconstructed source activity was then characterised with dynamic causal modelling (DCM). Eight models were compared for each patient, to evaluate the relative contribution of changes in intrinsic (excitatory and inhibitory) connectivity and endogenous afferent input. Bayesian model comparison established a role for changes in both excitatory and inhibitory connectivity during seizure activity (in addition to changes in the exogenous input). Seizures in both patients were associated with a sequence of changes in inhibitory and excitatory connectivity; a transient increase in inhibitory connectivity followed by a transient increase in excitatory connectivity and a final peak of excitatory–inhibitory balance at seizure offset. These systematic fluctuations in excitatory and inhibitory gain may be characteristic of (anti NMDA-R encephalitis) seizures. We present these results as a case study and replication to motivate analyses of larger patient cohorts, to see whether our findings generalise and further characterise the mechanisms of seizure activity in anti-NMDA-R encephalitis.

## Highlights

• We characterised seizures in patient with anti-NMDA-R encephalitis using EEG.
• Dynamic causal modelling was used to estimate causes of seizure activity.
• Characteristic variation of excitatory–inhibitory balance during seizure activity.
• This variation was seen for seizures within and between patients.

## Introduction

Anti-N-methyl-d-aspartate receptor (NMDA-R) antibody encephalitis was discovered in 2007 in several females with ovarian teratoma presenting with psychiatric and dys-autonomous symptoms. The disease has also been described in the paediatric population. It is an autoimmune disease with auto-antibodies targeting the NMDA-R (Dalmau et al., 2011). A multicentre study in the UK identified anti-NMDA-R encephalitis in 4% of patients with encephalitis (Granerod et al., 2010), which usually develops through specific phases (Iizuka et al., 2008). The prodromal phase can entail headache, fever, nausea, vomiting, diarrhoea, or upper respiratory-tract symptoms. Within 2 weeks, patients develop psychiatric symptoms; ranging from cognitive impairment to psychosis with delusions and hallucinations. There is often a rapid disintegration of language. This disease often progresses with neurological symptoms that can include reduced consciousness, oro-lingual-facial dyskinesia, seizures and dysautonomia. At this stage, the patient often requires treatment in an intensive care unit. The frequency and intensity of seizures usually decrease as the disease evolves; however, changes in medication and sedation can trigger status epilepticus at any given point of the disease process (Dalmau et al., 2007). The disease is often associated with tumours in the adult population, most often ovarian teratomas in female adults. The cause of anti-NMDA-R encephalitis (particularly in the paediatric population), when not associated with tumours, is unknown (Florance et al., 2009). Treatment usually comprises immune therapy, together with removal of any coexisting tumour. Without treatment the disease can improve slowly over several years but relapses are not uncommon (Iizuka et al., 2008). Seizures are common: in a study of 100 patients with NMDAR-encephalitis 76 had seizures. Most commonly, generalised tonic–clonic seizures followed by focal seizures; however, six patients developed status epilepticus and two developed epilepsia partialis continua. EEG abnormalities are often seen in patients, most commonly increased delta activity and monomorphic appearance of rhythmic delta activity (Dalmau et al., 2008). It is not uncommon to see “extreme delta brushes”, comprising rhythmic delta activity with a brush of beta activity (Schmitt et al., 2012). Electrographic seizure activity can sometimes be detected on the EEG.

Electrographic seizure activity is one of the most specific findings in clinical electroencephalography (Daly et al., 2002). However, the underlying pathophysiology is still poorly understood. Focal seizures typically exhibit three phases; initiation, propagation and termination (Schiff et al., 2000). They generally start with low-amplitude fast activity (Allen et al., 1992; Spencer et al., 1992; Alarcon et al., 1995; Wendling et al., 2003). This activity corresponds to the “ictal flattening”, sometimes seen before seizure onset in the scalp EEG. The cause of this pre-ictal pattern is thought to be caused by disinhibition of pyramidal neurons (Wendling et al., 2002), although other studies have suggested that *synchronization through inhibition* is important for the generation of low-amplitude fast activity at the onset of seizure activity (de Curtis and Gnatkovsky, 2009). During seizure propagation, there is usually a transition to large amplitude activity with slower oscillatory activity, together with spatial spreading. At this stage, seizure activity becomes more complicated — being mediated by a distributed epileptic network. Seizure offset usually entails a slowing of seizure activity and may be followed by a post ictal phase. Even seizure termination is governed by complex network dynamics that remains poorly understood. It has been suggested that seizures occur when there is an imbalance between excitatory and inhibitory conductance (Scharfman, 2007; Schiff and Sauer, 2008). Balanced excitation and inhibition in the brain is an important aspect of neuronal processing, enabling fast responses that require less energy consumption, and more efficient information processing (Sengupta et al., 2013; Sengupta and Stemmler, 2014). Active engagement of gain control mechanisms that maintain this balance may be compromised in epilepsy (Swann and Rho, 2014). However, it is unclear how this imbalance is related to seizure phenomenology in cortical circuits that generally exhibit normal excitatory–inhibitory balance (Soltesz, 2008). In this work, we use dynamic causal modelling with neural mass models to quantify excitation–inhibition balance in terms of intrinsic (within source) connectivity.

Neural mass models were first conceived by Wilson and Cowan using mean field theory to estimate the average activity of neuronal populations (Wilson and Cowan, 1972, 1973) based on the Hodgkin Huxley description of single neurons. Neuron mass models offer a computationally tractable model of mesoscopic neuronal activity. A particular useful variation of the Wilson and Cowan model was presented by Jansen and Rit, which has been used extensively in modelling different sorts of neuronal activity, including seizure activity (Jansen and Rit, 1995). The transition between normal and seizure activity has also been modelled in terms of bifurcations (qualitative changes in neural mass dynamics due to quantitative changes in model parameters) (Blenkinsop et al., 2012; Breakspear et al., 2006; Grimbert and Faugeras, 2006; Jirsa et al., 2014; Nevado-Holgado et al., 2012). However, multistability has also been proposed as an alternative to bifurcations (Benjamin et al., 2012; Lopes da Silva et al., 2003). Bifurcations are induced by changes in one or more parameters of the neural mass model. Parameter fluctuations during seizure onset has been inferred using a variety of methods, including Kalman filter techniques and genetic algorithms (Blenkinsop et al., 2012; Freestone et al., 2014; Nevado-Holgado et al., 2012; Schiff and Sauer, 2008; Ullah and Schiff, 2009, 2010; Wendling et al., 2005).

It is usually assumed that the transition from normal to seizure activity can be modelled with changes in connectivity between neuronal populations (Blenkinsop et al., 2012; Freestone et al., 2014; Wendling et al., 2002). Moreover, slow changes in ion concentrations have been shown, both experimentally and computationally, to induce rapid changes in neuronal dynamics that are formally similar to bifurcations (Bazhenov et al., 2004; Kager et al., 2000; Lewis and Schuette, 1975). Some modelling studies have considered glial cell ion homeostasis and conclude that changes in [K^+^] and [Na^+^] are necessary for seizure generation in hippocampal tissue (Ullah and Schiff, 2010). Similarly, the ability of extracellular oxygen to induce seizure activity has been verified in vivo and in computational models (Ingram et al., 2014; Wei et al., 2014). Furthermore, dynamical multi-stability has been used to simulate seizure activity, where [K^+^] can induce switching between (bistable) states, without the need for bifurcations (Frohlich et al., 2010). Finally, in contrast to mechanisms that are intrinsic to the source of seizure activity, several studies have highlighted the importance of multistability due to global changes in connectivity, causing both focal and general seizure activity (Benjamin et al., 2012; Terry et al., 2012).

In this paper, we characterise the evolution of seizure activity in terms of slow fluctuations in the (synaptic) connectivity among specific neuronal populations that constitute a canonical cortical microcircuit. Crucially, we evaluate these intrinsic changes, while allowing for concomitant changes in afferent activity from other distributed sources. To do this, we used dynamic causal modelling to analyse seizure activity in a patient with anti-NMDA-receptor encephalitis and attempt to replicate the findings in an identical analysis of a second patient.

Dynamic causal modelling (DCM) is a method for making inferences about the neuronal architectures that underlie measured time series, such as EEG (Friston et al., 2007). The main constituents of a DCM are a model of neuronal dynamics (e.g., a neural mass model) and a measurement model (e.g., a classical electromagnetic forward model). DCM has been widely used in neuroscience in modelling fMRI and EEG activity (David et al., 2006, 2008; Moran et al., 2008, 2011a,b,c; Friston et al., 2012). Crucially, several dynamic causal models can be inverted for any given data; enabling the evidence for competing models or hypotheses to be evaluated (with Bayesian model comparison). In contrast to alternative approaches (e.g., Kalman filtering), we apply DCM to spectral density measures of seizure activity. This enables one to (i) average spectral density measures over multiple seizures; (ii) estimate the spectral density of endogenous neuronal fluctuations with scale free (non-Markovian) temporal correlations and (iii) estimate any changes in these fast fluctuations during seizure onset. Furthermore, DCM allows one to explicitly parameterise the slow fluctuations in model parameters (e.g., excitatory and inhibitory connectivity) that contribute to the induction of seizure activity.

DCM has been used recently to model electrocorticography data during seizure onset. The authors found that changes in intrinsic connectivity were sufficient to explain seizure onset, and that seizure initiation was a result of transient loss of excitatory–inhibitory balance (Papadopoulou et al., 2015). This paper extends these findings with a more detailed analysis of non-invasive EEG data from human subjects with a known and specific pathophysiology of NMDA receptor function.

## Materials and methods

### Recordings

**Fig. 1:**
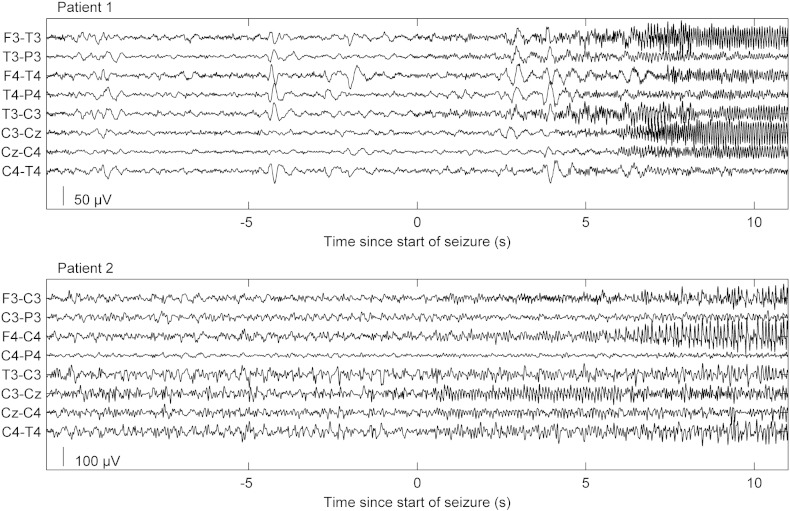
Seizure activity of patient 1 (top) and patient 2 (bottom). Note the focal start of seizure with relatively quick spreading between electrodes.

EEG recordings were obtained retrospectively from two female patients with anti-NMDA-R encephalitis from the database at Clinical Neurophysiology at Karolinska University Hospital, Stockholm, Sweden. Both patients were treated in intensive care with continuous EEG monitoring using nine scalp electrodes positioned according to the 10–20 system (F3, F4, C3, C4, Cz, P3, P4, T3 and T4) together with a reference electrode placed over Fz. The seizures recorded from patient 1 (age 19 years) started with 20 Hz activity, which reduced in frequency and increased in amplitude before termination after approximately 10–20 s, see Fig. 1. The seizures were clustered over time and occurred every two to three minutes. A total of 55 seizures free of artefacts were selected for modelling. The two seizures recorded from patient 2 (age 31 years) started with 10–15 Hz activity with a slow reduction in frequency before termination after approximately 60 s, see Fig. 1. We did not have access to higher density EEG recordings or invasive recordings as these patients had seizures only during the acute phase of the disease.

### Preprocessing

**Fig. 2:**
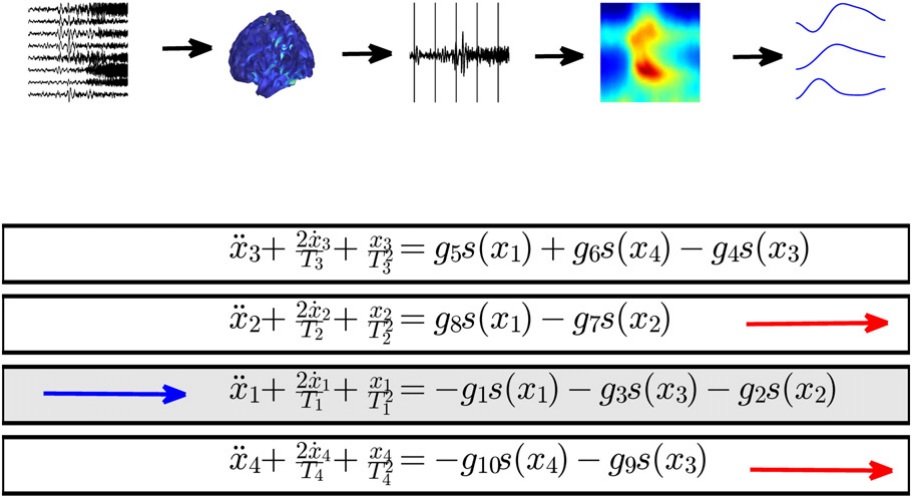
Schematic showing the steps of the analysis together with a schematic of the DCM used to generate the activity. Blue arrows depict afferent connections while the two red arrows show the cell populations generating the measured EEG signal.

After acquisition, the data was re-referenced to a common average and filtered using a bandpass filter (Butterworth 5th order filter) between 0.5 and 70 Hz. Line activity was removed using a notch filter at 50 Hz. We used an empirical Bayes beamformer to locate the source with the greatest spectral power during the first second of seizure activity in each patient (Belardinelli et al., 2012). We then reconstructed source activity and this location for further analysis. The time series for each seizure was divided into 2000 ms windows without overlap, for both patients. The size of the window was chosen as the maximum duration over which spectral activity remained approximately constant for each patient. More specifically, we used the maximum window length that retained 90% of the spectral power (as estimated using a complex Gaussian wavelet). The spectral densities of successive windows or epochs were estimated under a Bayesian multivariate autoregressive model, and the resulting spectral density averaged over seizures for each patient (referenced to the time of seizure onset). Based upon the resulting spectral density of seizure activity, we modelled fluctuations in spectral power between 1 Hz and 40 Hz with DCM, see Fig. 2.

### Dynamic causal modelling

The analysis described in the following sections used standard procedures for the Dynamic Causal modelling of spectral density (Moran et al., 2011a; Friston et al., 2012). For a formal description see Appendix A. DCM of (cross) spectral density has been validated in several contexts — and has been applied to ECoG data of seizure activity (Papadopoulou et al., 2015). Here, cortical activity was modelled using a neural mass model based on the canonical cortical microcircuit (CMC, Fig. 2), comprising four neuronal populations corresponding to granular, superficial pyramidal and deep pyramidal excitatory populations and inhibitory neurons (Moran et al., 2013). These neuronal populations are interconnected with inhibitory and excitatory (intrinsic) connections. Afferent connections terminated in the excitatory granular cells, which histologically would be equivalent to spiny stellate cells.

The canonical microcircuit was first described by Douglas and Martin (1991) based on the structure and function of the cat visual cortex. Structural and functional analysis of cat and rat neocortex from the visual and sensory–motor cortex has further supported the idea of a canonical microcircuit (Katzel et al., 2011; Thomson and Bannister, 2003; Yoshimura and Callaway, 2005) The CMC neural mass model is based on this and subsequent work, see (Bastos et al., 2012, 2015). The CMC model represents a minimal description of the cortical microcircuit, where neuronal dynamics are represented by generic inhibitory and excitatory cell populations; e.g., the effect of fast spiking interneurons rich in NMDA-R are represented by a generic inhibitory population (DeFelipe, 1999). In contrast to many neural mass models of cortical sources, the CMC includes two populations of excitatory pyramidal cells, located in superficial and deep cortical layers. These cell populations are the sources of forward and backward extrinsic (between-source) connections respectively (Pinotsis et al., 2014). There is evidence that both deep and superficial pyramidal cells are necessary to model the full spectrum of cortical activity, where faster activity is generated by the superficial population and slower activity by the deep population (Bastos et al., 2012; Buffalo et al., 2011; Roopun et al., 2006, 2008). In DCM, electrophysiological measurements such as the EEG, are modelled as a mixture of depolarisations in superficial and deep pyramidal cells, where, a priori, the contribution of deep pyramidal cells is optimised with a free parameter in relation to the (predominant) contribution from superficial populations.

Formally, the generative model of neuronal activity comprised eight coupled non-linear first order ordinary differential equations with delays (see Appendix A). This model is similar to that of Jansen and Rit (Jansen and Rit, 1995) but augmented to include four populations per source (and anatomically plausible intrinsic connectivity among the sources). The parameters of DCMs include intrinsic connection strengths, synaptic time constants, delays and parameters of the activation functions relating mean depolarisation to firing rates.

NMDA-R antibodies have been shown to target NMDA-R throughout the cortex – affecting both excitatory and inhibitory neurons – with a decrease in inhibitory synaptic density on excitatory neurons (Moscato et al., 2014). To model this, we equipped our with separate excitatory and inhibitory gain parameters, where gain corresponds to the sensitivity of a neuronal subpopulation to excitatory or inhibitory input. Crucially, the intrinsic connections from the inhibitory population were allowed to change during seizure onset and their evolution was modelled using a discrete cosine basis set with 8 components (over successive time windows of seizure activity, see Appendix A). These connections were chosen to model GABAergic tone; noting that certain fast spiking inhibitory interneurons such as chandelier cells and basket cells express NMDA receptors and preferentially target the source of EEG signal (principally, the superficial pyramidal cells) (Goldberg et al., 2003; Kawaguchi and Kubota, 1993). Similarly, all three excitatory intrinsic connections were allowed to change during seizure activity (Table 1). Note that all parameters of the afferent input were allowed to change with time giving the model full flexibility in modelling non-local input (see Appendix A). The methods used in this study for estimating inhibitory and excitatory connectivity have been previously validated in several studies, where LFP recordings have been measured together with pharmacological manipulations or with micro-dialysis measurements of extracellular glutamate levels (Moran et al., 2011a,b).

### Model inversion and comparison

**Fig. 3:**
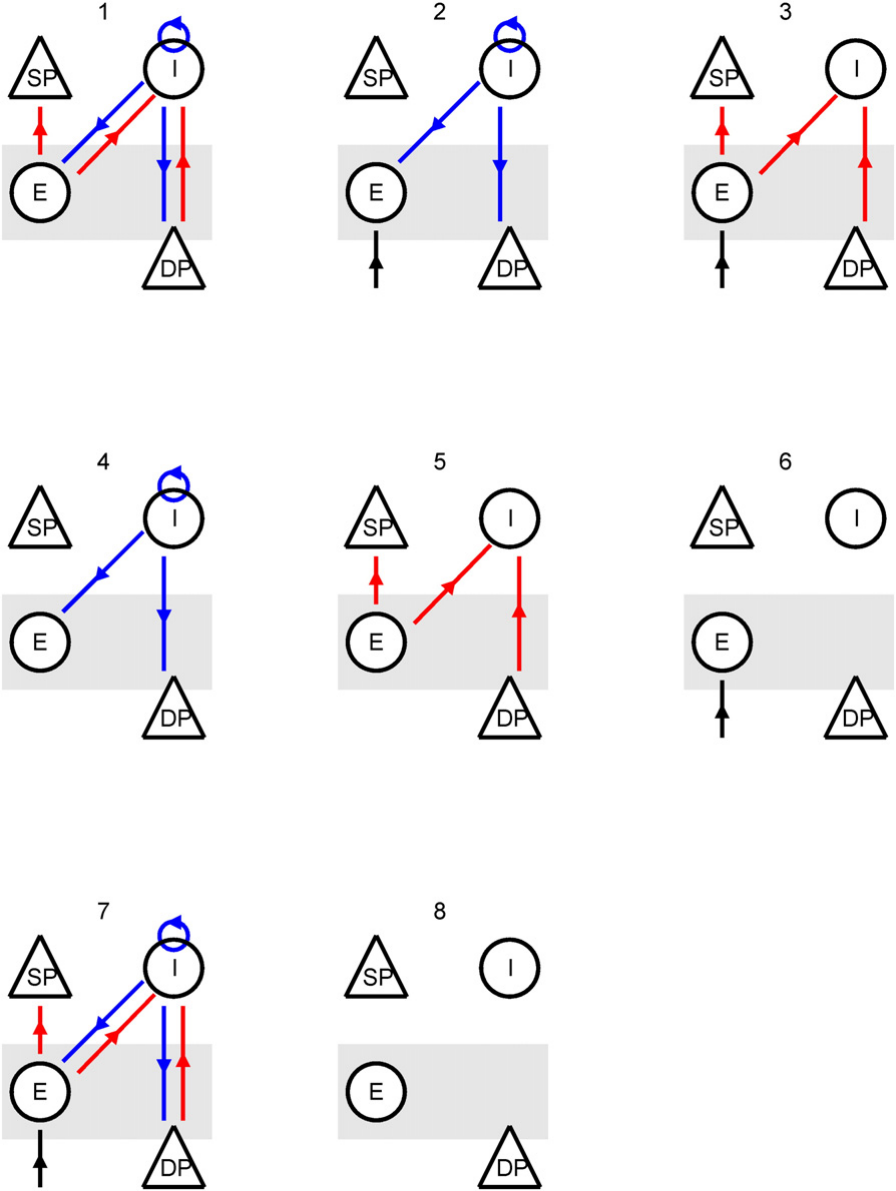
Eight models were inverted for each patient. Each source model comprised four neuronal populations representing different cell types from distinct cortical layers: excitatory granular cells, superficial and deep pyramidal cells and inhibitory interneurons. These cells were interconnected using 10 connections (not drawn in for clarity). Six of these connections were allowed to change over time to model changes in EEG spectra during the seizure. Only connections that were allowed to change are shown. All three connections from the inhibitory cells are marked in blue. All excitatory connections are marked in red. The spectral input marked in black was also allowed to vary in time. Model 7 – allowing changes in all three sets of parameters – was the most likely.

The parameters of the model were estimated following model inversion. The inversion was performed using a standard variational Bayesian scheme (Variational Laplace) under the Laplace approximation; i.e., the priors and posteriors were assumed to be Gaussian probability distributions (Friston et al., 2007). Effectively, inversion means finding the model parameters that maximise Bayesian model evidence. This automatically penalises complex models, because the model evidence comprises an accuracy and complexity term — the model with the greatest evidence is the simplest model that provides an accurate explanation for the data. Model inversion approximates the model evidence with a quantity called variational free energy (Appendix A; Friston et al., 2007). The variational free energy can then be used to compare competing models in terms of their probability is given the data. This is known as Bayesian model comparison (Stephan et al., 2010). Eight models were inverted (compared) for each patient, where different sets of parameters (intrinsic connections) were allowed to change over time — as shown in Fig. 3. These ranged from models in which nearly all connections could change (model 7) to a null model that precluded any changes over time windows (model 8). More specifically, we allowed for all possible combinations of changes in excitatory connections, inhibitory connections, and the parameters of the (power law) spectral density of afferent input from other sources.

### Summary

In summary, seizures were identified from the EEG recordings and epileptogenic sources were localised using a standard beamforming technique. The seizure activity at this source was used for subsequent DCM. The seizure activity was windowed and the spectral activity estimated in each window. We then used a neural mass model to generate the spectral activity of the underlying source by allowing slow fluctuations in excitatory and inhibitory (intrinsic) connections, while also allowing for changes in afferent activity from other parts of the brain. We used variational Laplace to estimate the ensuing model parameters for a set of models with and without changes in various parameters. The resulting model evidence was used to compare different models of seizure activity and identify the best explanation of observed seizure activity. Data preprocessing and modelling were performed using the academic freeware SPM12 (http://www.fil.ion.ucl.ac.uk/spm/).

## Results

**Fig. 4:**
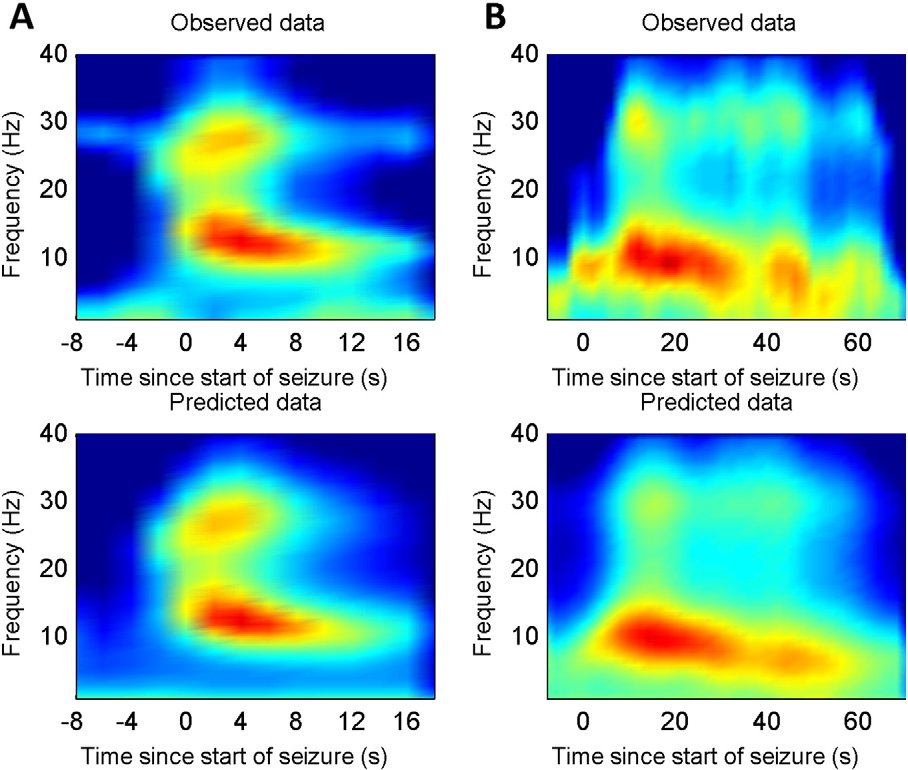
A). The top image illustrates the time frequency profile of observed seizure activity for patient 1 and the lower image illustrates the predicted time frequency plot after estimating the parameters of the DCM. Note the decrease in frequency as the seizure progresses. B). Similar illustration for patient 2. Note the difference in frequency content compared to patient 1 but the similar decrease in frequency over time.

The seizure activity of the first patient comprised low amplitude 20 Hz activity, which reduced in frequency to approximately 10 Hz but increased in amplitude. After 10–20 s the seizure terminated. Seizure activity was most prominent over the temporal region on the left side, although it showed a rapid and partial spread. In the second patient, seizure activity was manifest as 10–15 Hz activity with a slower reduction in frequency to about 5 Hz, accompanied by a slow increase in amplitude. The seizures terminated after approximately 60 s. Seizure activity was prominent over the central region but spread relatively quickly to several electrodes bilaterally. See Figs. 1 and 4 for time and time–frequency plots of seizure activity from the two patients.

Eight competing models were inverted for each patient; each allowing for different combinations of changes in inhibitory and excitatory intrinsic connectivity (and afferent input): see Fig. 3. The model with highest evidence (free energy) for both patients allowed for changes in both inhibitory and excitatory (i.e., GABA and NMDA dependent) connectivity together with changes in endogenous afferent input (i.e., the full model). The null models with no changes in model parameters were found to be least likely. Crucially, these results were exactly the same for both patients. The best model explained more than 97% of the variance in patient 1 and 95% in patient 2, see Table 2. The difference in log evidences (free energy) between the winning model and the next best model exceeded 10 in both cases. A difference of three is considered strong evidence in favour of the winning model (and corresponds to an odds ratio of about 20:1).

**Fig. 5:**
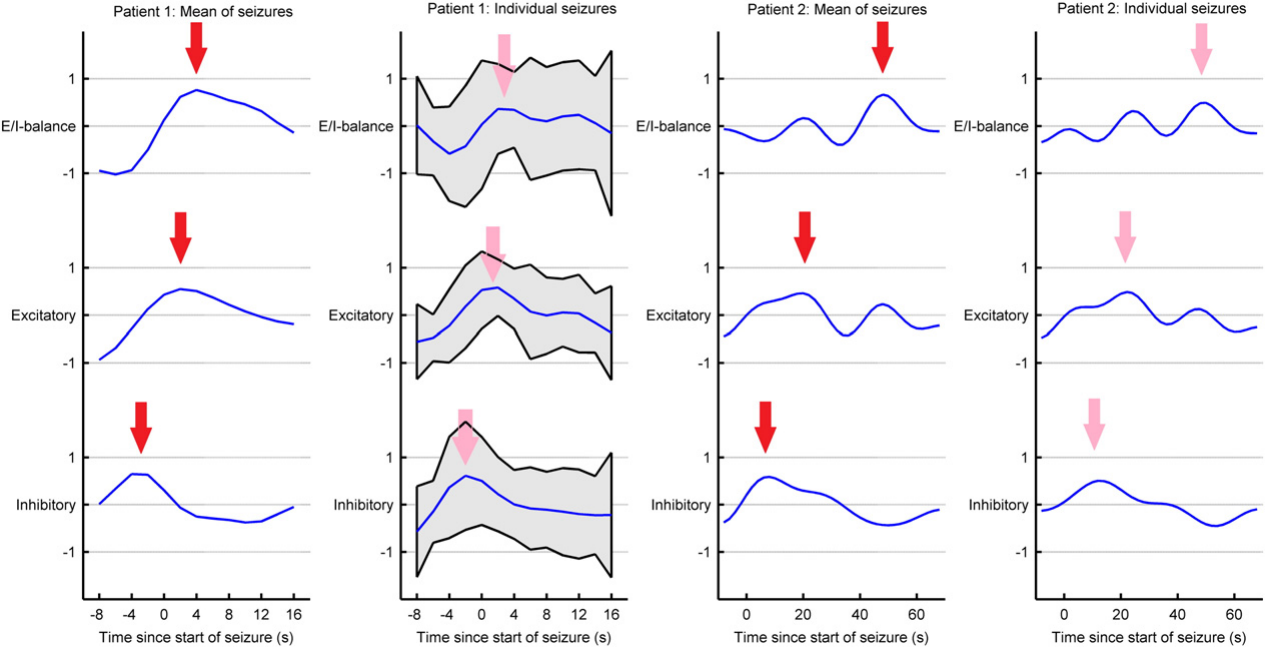
Changes in inhibitory and excitatory connectivity and their associated balance (difference). The parameters are shown as log scaling parameters. The first column shows changes inferred from average seizure activity and the second column shows the mean of connectivity changes (± 2 standard deviations) inferred from individual seizures for patient 1. The third and fourth column shows changes for patient 2, note that the standard deviation were not calculated for patient 2, as there were only two seizures. Note the peaks in activity occurring first for inhibitory activity followed by excitatory activity followed by excitatory-inhibitory balance (red and pink arrows).

**Fig. 6:**
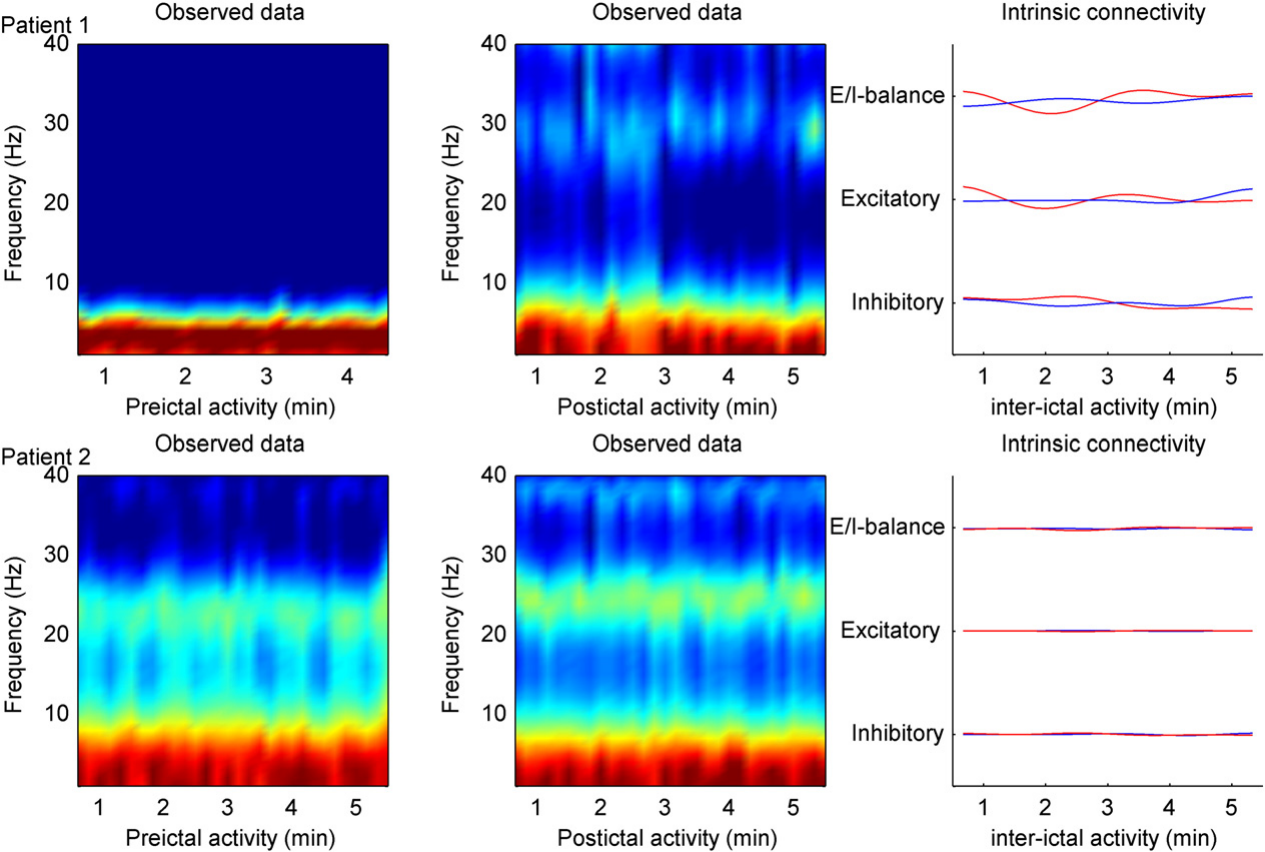
The top row illustrates electrographic activity for five minutes before and after seizure initiation for patient 1, together with inferred changes in connectivity (under the full model). The lower row shows similar results for patient 2. Note the absence of changes inferred during seizure activity.

The time course of inhibitory and excitatory connectivity and their balance (difference) showed systematic and similar changes during seizure activity; see Fig. 5. Seizure onset was induced by a transient increase in inhibitory connectivity followed by a transient increase in excitatory activity and a final peak in excitatory–inhibitory balance during seizure termination. Inversion of individual seizures showed similar results to the inversion of the averaged seizure (time frequency) activity. We also characterised source activity during 5 min of pre- and post-seizure activity, where the EEG showed no clear spectral changes and where the fluctuations in intrinsic connectivity (estimated under the full model) were almost negligible: see Fig. 6.

**Fig. 7:**
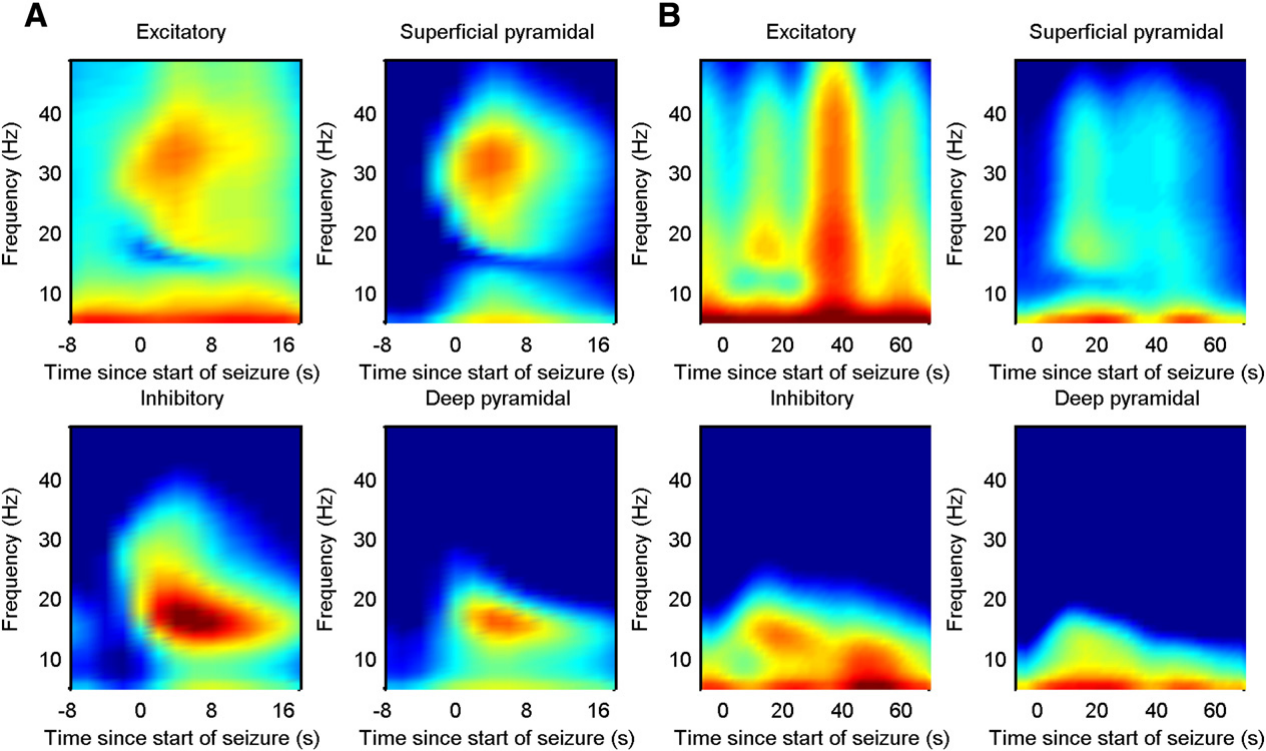
A). Time–frequency responses of each cell type in the canonical microcircuit model for patient 1. There is a general increase in activity of all four cell types during seizure activity. Superficial pyramidal cells show an early increase in spectral activity, with a subsequent slower reduction; while the inhibitory and deep pyramidal cells show a more sustained increase. B). Similar results for Patient 2 show a similar transient increase in superficial pyramidal activity and a more sustained response in the inhibitory cells.

**Fig. 8:**
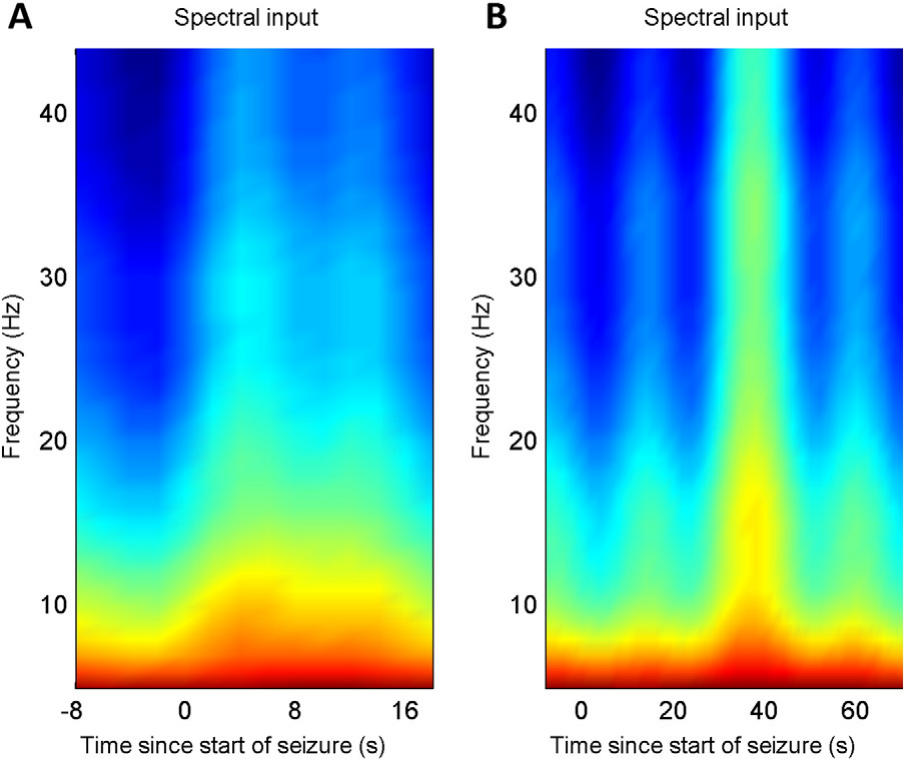
A). Spectral input for patient 1. Note that there is an increase in spectral activity during the seizure. B). Similar results for Patient 2 shows an increase in spectral activity towards the end of the seizure.

The overall effects of these changes in synaptic efficacy correspond to a disruption of excitatory–inhibitory balance during the seizure. To quantify the effect on each of the four populations, the predicted spectral activity of each population was reconstructed under the expected parameters of the best model. The ensuing time frequency response is shown in Figs. 7A and B, for the first and second patient respectively. It can be seen that there was an increase in spectral activity of all cell types during the seizure, with the superficial pyramidal cells showing a transient increase within a broad frequency range at seizure onset, while the inhibitory and deep pyramidal cells showed activity within a more narrow frequency range, which decreased in average frequency as the seizure progressed. In the winning model, the input to the cortical source (a summary of all subcortical and cortical afferents), was modelled as coloured noise (with spectral features) and was also allowed to change during the seizure activity. The time frequency profile of this afferent input is illustrated in Figs. 8A and B. It can be seen that there was an increase in the amplitude and a change in the spectrum of afferent input during the seizure, suggesting a distributed epileptogenic process beyond the source that was modelled explicitly.

### Summary

In summary, we found remarkable similarities between the explanations for seizure activity in both patients, regarding changes in the underlying cortical intrinsic connectivity. In general, these changes involved increases in the amplitude and changes in the spectrum of afferent (endogenous) activity, together with dissociable changes in inhibitory and excitatory intrinsic connectivity and their balance; peaking successively during seizure activity. Furthermore, model comparison revealed the contribution of changes in both excitatory and inhibitory connectivity — in addition to the afferent input from subcortical and cortical structures; suggesting the importance of distributed network dynamics in seizure initiation and maintenance.

## Discussion

In effect, we used DCM as a virtual microscope to track the changes in cortical dynamics during epileptic seizures registered with scalp EEG from two patients with anti-NMDA-R encephalitis. This allowed us to infer activity of the constituent cell populations in epileptogenic sources — and the underlying changes in connectivity and afferent input subtending this activity. Our results indicated an increase in activity of all cell types during seizure activity. Furthermore, we identified systematic changes in inhibitory and excitatory connectivity — suggesting a disruption of excitatory–inhibitory balance during seizure activity. This is, to our knowledge, the first time that cortical dynamics of seizure activity induced by anti-NMDA encephalitis has been inferred quantitatively from non-invasive EEG data.

Increased disinhibition has been previously suggested as a possible cause of epileptic seizures (Wendling et al., 2002); however, several in-vivo and in-vitro studies have shown that there is sustained inhibitory activity during seizures and that disinhibition may not be necessary for seizure generation (Dailey et al., 1989). In this study, we see a more complex picture, where we found a sequential peaking of inhibitory connectivity, excitatory connectivity and finally excitatory–inhibitory balance. The net effect on each of the main four cell types within cortical sources suggest an increase in activity but a decreasing frequency of the deep pyramidal cells activity as the seizure progressed. The exact timing of the activation of each cell type is controlled by slow fluctuations in the intrinsic connections to and from the input (granular or spiny stellate population). At seizure onset, we see increased activity in the superficial pyramidal cells, which convey mainly fast (beta and low gamma band) activity together with a slightly prolonged activation of deep pyramidal cells, which show slower activity (alpha and theta band). This was more pronounced in the first patient, where there is a greater change in frequency during the seizure. This change of activation from superficial layers of the cortex to deeper (infragranular) layers might be a ubiquitous feature of seizure activity — as a decrease in the main oscillatory frequency is characteristic of seizure activity. This electrophysiological pattern has also been termed the brain chirp (Schiff et al., 2000).

We were able to ask, using Bayesian model comparison, whether seizure activity originating from the epileptogenic source depended on other regions of the brain or not. Model comparison suggested that seizure activity was indeed dependent on other subcortical or cortical regions. However, this time varying spectral input was not sufficient to explain seizure activity — as changes in intrinsic connectivity was necessary to explain changes in spectral activity (and the different activity profiles of superficial and deep pyramidal populations). These fluctuations in intrinsic connectivity can be thought of as changes in synaptic gain. Changes in gain can be mediated by various biophysical and biochemical mechanisms, such as membrane-potential dependent ion channels conductance or changes in ion concentrations. The effects of intra and extracellular ion concentrations have been associated with the generation of seizures in vivo and in vitro (Frohlich et al., 2010; Ingram et al., 2014). Experimental and computational studies speak to the importance of ionic homeostasis and several models have included glial cell physiology in this context (Frohlich et al., 2010; Ullah et al., 2009). As epileptic seizures are sometimes prolonged – and can last for minutes or hours – there is also a possible role for short and long-term receptive plasticity. Furthermore, seizures are not physiological events and may affect neuronal dynamics pathologically through energy (ATP) or oxygen depletion, which has been shown in vivo (Ingram et al., 2014; Wei et al., 2014). In short, further modelling of ionic and synaptic homeostasis might be necessary to understand the detailed causes of the slow parameter variations of the sort seen in the study.

Several studies have shown that it is possible to estimate synaptic parameters using inversion schemes based on extended and unscented Kalman filters but also using alternative methods like multi-objective genetic algorithms (Freestone et al., 2014; Nevado-Holgado et al., 2012; Schiff and Sauer, 2008; Ullah and Schiff, 2010). It is important to appreciate that the DCM scheme used here is formally distinct from filtering schemes. This is because the data features used by DCM here are not the timeseries but their spectral density. Effectively, this enables DCM to parameterise the second order statistics of endogenous fluctuations generating seizure activity. In our case, we used a mixture of scale free dynamics (specified with a 1/f form in frequency space) and other coloured components we attribute to fast neuronal fluctuations in afferent input from other sources. This means that we do not have to estimate hidden neuronal states (as in Bayesian filtering) but can formulate the inverse problem purely in terms of unknown model parameters. Furthermore, inverting models based upon spectral responses enables one to average over multiple seizures to provide a computationally efficient summary of seizure activity.

Biological considerations suggest dysfunction in both excitatory and inhibitory synaptic activity, as these synaptic connections are affected in anti NMDA-R encephalitis (Moscato et al., 2014). We therefore modelled fluctuations in the intrinsic gain of excitatory and inhibitory connectivity. It is possible that several excitatory (and inhibitory) connections could change independently during seizure onset; in principle, this can be addressed using Bayesian model comparison. Note that introducing more free (changes in intrinsic connectivity) parameters would increase the accuracy of the fits but may reduce model evidence by incurring a large complexity cost. In this paper, we used a parsimonious model of gain fluctuations, because our focus was on changes in intrinsic connectivity, relative to changes in extrinsic afferents.

In this study we focused on the temporal dynamics of seizure activity but not its spatial dynamics. The (pragmatic) reason for this was the sparse set of electrodes used for recording the seizure. We used a low-density setup, as these patients were being treated in an intensive care unit and the clinical indication for EEG was to detect the presence of seizure activity (not source localisation). We were able, by visual analysis of the EEG, to determine that the seizures were focal. This observation was used to motivate a single (epileptogenic) source model. The main reason for reconstructing distributed seizure activity (including source localisation) is to delineate the seizure onset zone in cases of pre-surgical evaluation of patients with refractive epilepsy. Usually this is accomplished with high-density recordings. In the future, we hope to model seizure activity recorded with high density EEG and apply similar methods using DCM to obtain a more comprehensive characterisation of the spatiotemporal dynamics.

Recently, a canonical (Epileptor) model of epileptic seizure activity was presented, which provided a formal taxonomy of seizure activity in terms of bifurcations (Jirsa et al., 2014; Proix et al., 2014). The onset and offset of seizure activity were described as saddle node and homoclinic bifurcations, respectively. Predictions of inter spike timing and direct current shifts associated with seizure onset and offset were also confirmed in vitro. This Epileptor model uses coupling between dynamics at different time scales — such that bifurcations can be induced by slowly varying neuronal states. In the present study no dynamical model was used for the slow fluctuations of synaptic parameters, only smoothness constraints were imposed. In a forthcoming study we will consider biophysically plausible DCMs in which slow parametric variations depend on the fast activity of the hidden states, based on activity dependent plasticity.

We assumed fluctuations in synaptic efficacy were at least one timescale slower than EEG activity. This prior assumption was imposed using smooth temporal (discrete cosine) basis functions to model changes in parameters (see Appendix A). This procedure requires the whole data set to be inverted at once, which is computationally intensive but practically possible, if a small number of cortical sources are modelled. When analysing a more realistic dataset (with several sources of an epileptic network) model inversion can become computationally intractable. In this setting, Bayesian belief updating, may provide a more efficient scheme for estimating slowly varying parameters, where estimates are updated from epoch or window to window. This form of updating is formally similar to the Kalman filter techniques used in other studies to infer parameter dynamics from non-invasive and invasive recordings (Schiff and Sauer, 2008; Ullah and Schiff, 2010). If the generative model is very non-linear other techniques can be used such as genetic algorithms or numerical continuation procedures for bifurcations analysis (Blenkinsop et al., 2012; Nevado-Holgado et al., 2012). Finally, Generalised Filtering is a recently described Bayesian filtering scheme that allows for estimation of slowly varying parameters, which has already been used to estimate hidden states and parameters in fMRI analysis (Friston et al., 2010). In a forthcoming study, we will compare the analysis described above with computationally efficient Bayesian belief updating schemes. These might provide accurate and efficient schemes for the inversion of larger epileptic networks.

## Conclusion

With DCM we were able to infer the cortical pathophysiology of seizure activity from two patients with anti-NMDA-R encephalitis recorded in a clinical setting. We found distinctive changes in excitatory–inhibitory balance were necessary to explain observed seizure activity and that these changes were conserved over seizures. The same pattern of changes was observed in a second patient with the same seizure aetiology. We hope to model an extended group of patients to see whether our findings generalise and further characterise the mechanisms of seizure activity in anti-NMDA-R encephalitis.

## Funding

This work was supported by the Wellcome Trust (088130/Z/09/Z) and a postdoctoral scholarship from the Swedish Brain Foundation (Hjarnfonden, PS2013/0017) to GC. BS and KJF are funded by a Wellcome Trust Principal Research Fellowship (088130/Z/09/Z).

## Figures and Tables

**Table 1:** (Free) Parameters estimated by dynamic causal modelling. The second column describes the prior values and the third the log variances.

Parameters estimated	Notation	Prior mean	Log prior variance
*Constant parameters*			
Time constants (Hz)	*T_i_*, *i* = 1, …, 4	[0.25 0.17 0.08 0.07] ∗ 1000	0.0625
Connectivity constants (Hz)	*g_i_*, *i* = 1, 2, 7, 10	[0.8, …, 0.2] ∗ 1000	0.0625
Slope of sigmoid function	*γ*	0.67	0.03125
Time delay for connections (ms)	*d*	1	0.03125
			
*Time dependent parameters*			
Connectivity parameters			
Inhibitory (Hz)	*g*_3_(*t*)	1.6 ∗ 1000	0.0625
Inhibitory (Hz)	*g*_4_(*t*)	0.8 ∗ 1000	0.0625
Inhibitory (Hz)	*g*_9_(*t*)	0.4 ∗ 1000	0.0625
Excitatory (Hz)	*g*_5_(*t*)	0.8 ∗ 1000	0.0625
Excitatory (Hz)	*g*_6_(*t*)	0.4 ∗ 1000	0.0625
Excitatory (Hz)	*g*_8_(*t*)	0.8 ∗ 1000	0.0625
Endogenous spectral input			
Amplitude of spectral density of input	*a*_1_(*t*)	1	0.0078125
Power law exponent of spectral density of input	*a*_2_(*t*)	1	0.0078125
Amplitude of spectral density of measurement noise	*b*_1_(*t*)	1	0.0078125
Power law exponent of spectral density of measurement noise	*b*_1_(*t*)	1	0.0078125
Spectral innovation of input	*d_i_*(*t*), *i* = 1, …, 8	1	0.0078125

**Table 2:** The variance described and the free energy for the different models inverted for each patient. Note that the winning model (highest free energy) also had the best fit and these were the same models for both patients. The free energies are expressed relative to the null model.

Model	Patient 1		Patient 2	
Variance explained	Free energy	Variance explained	Free energy
Inhibitory + excitatory + endogenous	0.97	1430	0.95	1740
Inhibitory + excitatory	0.97	1380	0.94	1650
Inhibitory + endogenous	0.97	1320	0.94	1600
Excitatory + endogenous	0.96	1310	0.94	1680
Inhibitory	0.90	860	0.91	1130
Excitatory	0.91	1010	0.92	1460
Endogenous	0.91	950	0.91	1230
Null	0.50	0	0.75	0

## Appendix A

### A.1 Canonical microcircuit model

The canonical microcircuit represents the activity of a cortical source and comprises four populations of cells that are assigned to various layers in the cortex. Each cell type is represented by a critically damped harmonic oscillator driven by connections from other populations within the source and through extrinsic connections from other sources (with a delay for transmission between cell types, *δ*), see Fig. 2. The neuronal dynamics are given by,

A1.1 $$\frac{d^{2}\vec{X}}{dt^{2}}+2T^{-1}\frac{d\vec{X}}{dt}+T^{-2}\vec{X}=\;\vec{U}+g\vec{S}\left(\vec{X}\right)$$

• $\vec{X}$ is a 4 × 1 vector where the *i*-th element represents the mean potential of the *i*-th population.
• **T** is a 4 × 4 diagonal matrix containing the time constants of the four populations.
• $\vec{U}$ is a 4 × 1 vector of afferent input to the *i*-th population *δ* on: here, just the first.
• **g** is a 4 × 4 matrix of intrinsic connections along the four populations, excitatory connections are positive while inhibitory connections are negative.
• $\vec{S}\left(\vec{X}\right)$ is a 4 × 1 sigmoid vector function of mean potential that models the firing rate of the population; i.e.,
  A1.2 $$S_{i}\left(\vec{X}\right)=\mathrm{Sigm}\left(\vec{X}\left(t\right)\right)=\tanh\left(\gamma \vec{X}\left(t-\delta \right)\right)$$

Where *δ* models the conduction delays and *γ* models the sensitivity of neuronal firing to depolarisation.

See Table 1 for definition of parameters. In the current model, ten of the intrinsic connectivity gains *g_ij_* were nonzero. The predicted response, *y* (the activity of the virtual electrode), is given by:

A1.3 $$y=C^{T}\vec{X}$$

where **C** is 4 × 1 vector describing the lead field of each subpopulation in the cortex, in the current model, **C** is a weighted sum of the membrane potentials of the two pyramidal cell populations.

### A.2 Transfer functions

We are interested in modelling the perturbations of this system around its fixed point induced by exogenous input. Under local linear assumptions, one can write Eq. (A1.1) in normal form; i.e., as a linear first order differential system:

A2.1 $$\frac{d\vec{X}}{dt}\;=J\vec{X}+\vec{U}$$

**J** is the Jacobian of the operator mapping the neuronal states to their time-derivatives and $\vec{X}$ is now a 8 × 1 vector. The Laplace transform of Eqs. (A1.3) and (A2.1), together a re-arrangement furnishes the transfer function mapping the exogenous input to the spectral output of the source.

A2.2 $$\tilde{y}=\;H\left(s\right)=C^{T}{\left(sI-J\right)}^{-1}\tilde{\vec{U}}$$

The tilde sign is used to denote the Laplace transform. The transfer function *H*(*s*) will depend on the parameters of the model through the Jacobian **J** and the parameterised exogenous input, which means that the spectral output is a function of model parameters *θ*.

A2.3 $$\tilde{y}=H\left(\theta ,s\right)$$

See Moran et al. (2011a,b,c) and Friston et al. (2012) for further details. The spectral density of the input is modelled as 1/f noise with amplitude and power law parameters together with a parameterised spectral innovation. The spectral innovation was parameterised using a discrete cosine set of order 8. Channel-specific measurement noise was modelled as 1/f noise with amplitude and power law parameters.

### A.3 Changes in parameters

Three of the (*g*_3_, *g*_4_, *g*_9_) representing inhibitory connections and three excitatory connections (*g*_5_, *g*_6_, *g*_8_) were allowed to change with time, as were the parameters of the (power law) spectral density of afferent input, see Table 1. As we wanted to infer slow changes over time we approximated this variation using a truncated series of cosine functions.

A3.1 $$g_{i}\left(t\right)=\;g_{i}\Gamma_{inh}\left(t\right),\;i=3,4,9$$

A3.2 $$g_{i}\left(t\right)=\;g_{i}\Gamma_{exc}\left(t\right),\;i=5,6,8$$

The time course of changes Γ*_i_*(*t*), *i* = *inh*, *exc*, was parameterised as a discrete cosine set of order 8. Similar equations were used for parameters determining the spectral input.

### A.4 Model inversion and variational free energy

Model inversion corresponds to optimising variational free energy with respect to an approximate posterior over the unknown model parameters. For more details on the following see (Friston et al., 2007). Variational free energy, *F*, is defined as the difference between the evidence for a model and the Kullback–Leibler divergence (*D_KL_*) between the approximating posterior, *q*(*θ*), and the true posterior.

A4.1 $$F\left(q\right)=\;\log p\left(\tilde{y}|m\right)-D_{KL}\left(q\left(\theta \right)||p\left(\theta |\tilde{y}\right)\right)$$

Note that for any pair of probability distributions, *q* and *p*, *D*_*KL*_(*q*||*p*) ≥ 0, with equality only when the distributions are identical. The free energy evaluated at any given *q* is less than the evidence for the model. This means that by maximising *F* with respect to the approximate posterior, one can evaluate a lower bound for the evidence, while (at the same time) minimising the difference between the approximate and true posteriors. Note that the sign of the variational free energy used here is opposite to the definition used in the physics literature.

### A.5 Variational Laplace

The free energy can be written as the difference between the expected energy and the entropy of the variational parameter distribution.

A5.1 $$F=\;{\langle \log\left(p\left(\tilde{y}|\vec{\theta }\right)\right)\rangle }_{q}+\;{\langle \log\left(p\left(\vec{\theta }\right)\right)\rangle }_{q}-\;{\langle \log\left(q\left(\vec{\theta }\right)\right)\rangle }_{q}\text{.}$$

The first two terms represent the expected energy. Under the Laplace assumption that the approximate posterior is Gaussian with mean and covariance (*μ*, Σ), the expected energy can be expressed as follows (ignoring constants).

A5.2 $$L\left(\mu \right)=\;-\frac{1}{2}\varepsilon^{T}\Pi \varepsilon -\frac{1}{2}{\varepsilon^{\theta }}^{T}\Pi^{\theta }\varepsilon^{\theta }+\;\frac{1}{2}\log\left|\Pi \right|$$

• *μ* is the mean of the approximate posterior
• *ε* = *ỹ*(*s*) − *H*(*μ*, *s*) is the prediction error
• *ε*^*θ*^ = *μ* − *μ*^*θ*^ is the difference between the posterior and prior mean.
• Π is the precision of sampling errors
• Π^*θ*^ is the precision (inverse covariance) of the prior distribution.

The free energy is maximised by performing a gradient ascent on Eq. (A5.2). At convergence the mean of the posterior is retrieved. Finally, the posterior covariance is estimated from the Hessian of the inverse energy at its mean.

A5.3 $$\sum_{ij}^{-1}=-\frac{\partial^{2}L\left(\mu \right)}{\partial \theta_{i}\partial \theta_{j}}$$
